# Supplementary material for: Microalgae to Bioenergy: Optimization of Aurantiochytrium sp. Saccharification
Source: Biology (Basel). 2023 Jun 29;12(7):935. doi: 10.3390/biology12070935 (PMC10376672; doi:10.3390/biology12070935)
Supplement: Supplementary file 1 [file biology-12-00935-s001.zip › biology-2398401-supplementary.pdf]

**Supplementary material**

**Table S1.** Variance analysis for the response surface reduced model for sugar concentration ($y_1$)

| **Source** | **Degree of freedom** | **Sum of Squares** | **Mean Square** | ***F-value*** | ***p*** |
|---|---|---|---|---|---|
| Model | 7 | 580.87 | 82.98 | 340.41 | < 0.001 |
| $x_1$ | 1 | 28.62 | 28.62 | 117.43 | < 0.001 |
| $x_2$ | 1 | 539.80 | 539.80 | 2214.37 | < 0.001 |
| $x_3$ | 1 | 3.76 | 3.76 | 15.41 | 0.001 |
| $x_2^2$ | 1 | 2.20 | 2.20 | 9.04 | 0.006 |
| $x_1 x_2$ | 1 | 1.15 | 1.15 | 4.70 | 0.040 |
| $x_1 x_3$ | 1 | 2.72 | 2.72 | 11.16 | 0.003 |
| $x_2 x_3$ | 1 | 2.62 | 2.62 | 10.77 | 0.003 |
| Residual | 24 | 5.85 | 0.24 | | |
| Lack-of-fit | 7 | 1.51 | 0.22 | 0.85 | 0.565 |
| Pure Error | 17 | 4.34 | 0.26 | | |
| Corrected Total | 31 | 586.72 | | | |
| $R^2$ = 0.990; adjusted $R^2$ = 0.987; predicted $R^2$ = 0.981 | | | | | |

**Table S2**. Variance analysis for the response surface reduced model for yield ($y_2$)

| **Source** | **Degree of freedom** | **Sum of Squares** | **Mean Square** | ***F-value*** | ***p*** |
|---|---|---|---|---|---|
| Model | 6 | 48.44 | 8.07 | 29.45 | < 0.001 |
| $x_1$ | 1 | 36.08 | 36.08 | 131.62 | < 0.001 |
| $x_2$ | 1 | 0.69 | 0.69 | 2.50 | 0.126 [a] |
| $x_3$ | 1 | 2.50 | 2.50 | 9.11 | 0.006 |
| $x_2^2$ | 1 | 3.09 | 3.09 | 11.26 | 0.003 |
| $x_1 x_2$ | 1 | 2.29 | 2.29 | 8.35 | 0.008 |
| $x_1 x_3$ | 1 | 3.80 | 3.80 | 13.85 | 0.001 |
| Residual | 25 | 6.85 | 0.27 | | |
| Lack-of-fit | 8 | 2.65 | 0.33 | 1.34 | 0.289 |
| Pure Error | 17 | 4.20 | 0.25 | | |
| Corrected Total | 31 | 55.29 | | | |
| $R^2$ = 0.876; adjusted $R^2$ = 0.846; predicted $R^2$ = 0.794 | | | | | |

[a] Statistically insignificant parameter on a 95% confidence level. However, it was kept in the reduced model for hierarchic reasons.
